# MAT2A Localization and Its Independently Prognostic Relevance in Breast Cancer Patients

**DOI:** 10.3390/ijms22105382

**Published:** 2021-05-20

**Authors:** Pei-Yi Chu, Hsing-Ju Wu, Shin-Mae Wang, Po-Ming Chen, Feng-Yao Tang, En-Pei Isabel Chiang

**Affiliations:** 1 School of Medicine, College of Medicine, Fu Jen Catholic University, New Taipei 242, Taiwan; chu.peiyi@msa.hinet.net; 2 Department of Pathology, Show Chwan Memorial Hospital, Changhua 500, Taiwan; 3 Department of Health Food, College of Health, Chung Chou University of Science and Technology, Changhua 510, Taiwan; 4 National Institute of Cancer Research, National Health Research Institute, Tainan 704, Taiwan; 5 Department of Biology, National Changhua University of Education, Changhua 500, Taiwan; hildawu09@gmail.com; 6 Research Assistant Center, Show Chwan Memorial Hospital, Changhua 500, Taiwan; yaoming9@yahoo.com.tw; 7 Department of Medical Research, Chang Bing Show Chwan Memorial Hospital, Lukang Town, Changhua County 505, Taiwan; 8 Department of General Surgery, Show Chwan Memorial Hospital, Changhua 500, Taiwan; wangznmg@gmail.com; 9 Department of Food Science and Biotechnology, National Chung Hsing University, Taichung 40402, Taiwan; 10 Biomedical Science Laboratory, Department of Nutrition, China Medical University, Taichung 40402, Taiwan; vincenttang@mail.cmu.edu.tw; 11 Innovation and Development Center of Sustainable Agriculture (IDCSA), National Chung Hsing University, Taichung 40402, Taiwan

**Keywords:** breast cancer, GNMT, MAT1A, MAT2A, subcellular localization, prognosis

## Abstract

(1) Background: methionine cycle is not only essential for cancer cell proliferation but is also critical for metabolic reprogramming, a cancer hallmark. Hepatic and extrahepatic tissues methionine adenosyltransferases (MATs) are products of two genes, MAT1A and MAT2A that catalyze the formation of *S*-adenosylmethionine (SAM), the principal biological methyl donor. Glycine N-methyltransferase (GNMT) further utilizes SAM for sarcosine formation, thus it regulates the ratio of SAM:S-adenosylhomocysteine (SAH). (2) Methods: by analyzing the TCGA/GTEx datasets available within GEPIA2, we discovered that breast cancer patients with higher *MAT2A* had worse survival rate (*p* = 0.0057). Protein expression pattern of MAT1AA, MAT2A and GNMT were investigated in the tissue microarray in our own cohort (n = 252) by immunohistochemistry. MAT2A C/N expression ratio and cell invasion activity were further investigated in a panel of breast cancer cell lines. (3) Results: GNMT and MAT1A were detected in the cytoplasm, whereas MAT2A showed both cytoplasmic and nuclear immunoreactivity. Neither GNMT nor MAT1A protein expression was associated with patient survival rate in our cohort. Kaplan–Meier survival curves showed that a higher cytoplasmic/nuclear (C/N) MAT2A protein expression ratio correlated with poor overall survival (5 year survival rate: 93.7% vs. 83.3%, C/N ratio ≥ 1.0 vs. C/N ratio < 1.0, log-rank *p* = 0.004). Accordingly, a MAT2A C/N expression ratio ≥ 1.0 was determined as an independent risk factor by Cox regression analysis (hazard ratio = 2.771, *p* = 0.018, n = 252). In vitro studies found that breast cancer cell lines with a higher MAT2A C/N ratio were more invasive. (4) Conclusions: the subcellular localization of MAT2A may affect its functions, and elevated MAT2A C/N ratio in breast cancer cells is associated with increased invasiveness. MAT2A C/N expression ratio determined by IHC staining could serve as a novel independent prognostic marker for breast cancer.

## 1. Introduction

In the US, the cancer incidence had been stable in women and declined by approximately 2% per year in men (2006 to 2015). The cancer death rate decreased annually by 1.4% and 1.8% (2007 to 2016), respectively [1]. Breast cancer is the most diagnosed cancer type among the gynecologic cancers [2], and it is the second leading cause of cancer death in the US [3]. The breast cancer incidence rate increased slightly by 0.3% per year (2012 to 2016) due to rising rates of local stage and hormone receptor-positive diseases in the US. In 2012, breast cancer was the most common type of cancer among females in the Asia-Pacific region, accounting for 18% of all cases, and was the fourth most common cause of cancer-related deaths (9%). Rapid rises were observed in several Asian countries, and the incidence rates of breast cancer in developing countries throughout the Asia-Pacific region are anticipated to continue to increase [4].

Breast cancer usually displays frequent intra- and inter-tumor heterogeneity presenting genetic and non-genetic alterations that often promote the progression of cancer cells [5,6,7]. Although estrogen receptor (ER), progesterone receptor (PR), and epidermal growth factor receptor 2 (HER2) are currently used in the clinic for prognostic evaluation as well as to assort breast cancer patients for appropriately targeted therapies, treatment failure often occurs in triple-negative breast cancer (TNBC) that accounts for approximately 15–20% of breast cancer patients [8]. The distinctly aggressive common nature of TNBCs includes higher rates of relapse and shorter overall survival. Breast tumor belongs to a heterogeneous group without well-defined molecular target therapies; and exploring novel disease markers as well as molecular targets for developing future therapies is needed.

Folate-mediated one-carbon metabolism is essential for methylation status [9] and nucleotide biosynthesis [10,11,12]; both are critical in cancer development and therapeutics [13,14]. Methionine is converted to the cellular methyl donor, *S*-adenosylmethionine (SAM) through the transfer of adenosine from ATP to the methionine sulfur that is catalyzed by methionine adenosyl transferases (MATs). Mammals have three distinct forms of MAT (MATI, MATII and MATIII), encoded by two distinct genes (*MAT1A* and *MAT2A*). Among the MATs, *MAT1A* is mainly expressed in the liver, whereas *MAT2A* and *MAT2B* are widely expressed in non-parenchymal cells of the liver and extrahepatic tissues [15]. Accumulating evidence suggests that dysregulation of all three *MAT* genes plays a significant role in the development of gastrointestinal cancers including hepatocellular carcinoma, cholangiocarcinoma, tumors from colon, gastric, and pancreas tissues [15] as well as tumors derived from other tissues including breast and prostate. MATII consists of α2 catalytic subunit encoded by *MAT2A* and β regulatory subunit encoded by *MAT2B*. Hepatocellular carcinoma (HCC) is characterized by the low expression of the liver-specific *MAT1A* gene that encodes the SAM synthesizing isozymes MATI/III; and the high expression of the widely *MAT2A* that encodes the MATII isozyme and high expression of *MAT2B* that encodes a β-subunit without catalytic action, but it can regulate MATII enzymatic activity [15].

Wang et al. recently demonstrated that methionine cycle flux can specifically modulate the epigenetic state of cancer cells and drives tumor initiation [16]. Studies have underscored the role of *MAT* genes beyond the liver cancer development. In human colorectal cancer cell lines, inhibition of *MAT2A* and *MAT2B* by SAM or miR-34a/b expression inhibited tumor migration and invasion in vitro [17]. The tumor suppressor activity of miR-203 in HCC was proposed to be partially dependent on its inhibition of *MAT2A* and *MAT2B* [18]. These studies indicated that *MAT2A* and *MAT2B* could be important targets for inhibiting cancer metastasis.

A higher level of *MAT2B* has been found to be correlated with worse relapse-free survival in triple-negative breast cancer (TNBC) [19]. Induction of *MAT2A/MAT2B* confers growth and survival advantage to cancerous cells and enhancing tumor migration [15]; hence, understanding the role of *MAT* genes in tumorigenesis can help develop potential and effective strategies for cancer treatment and chemoprevention. Direct inhibition of *MAT2B* suppressed cell growth and migration and induced apoptosis in breast cancer cell MDA-MB-231 and MDA-MB-468 [19]. The basal expression level of *MAT2A* was upregulated in tamoxifen-resistant-MCF-7 cells [20]. These studies suggest that targeting *MAT* genes could be potential therapeutic intervention for TNBC and the role of MATs in human breast cancer needs further investigation.

Many transformed cells and embryonic stem cells are dependent on *MAT2A* to synthesize SAM and maintain their epigenome. The combination of methionine depletion and *MAT2A* inhibition has been used to suppress SAM biosynthesis and eradicate CD44hi/C24low cancer stem cell population. Methionine depletion induced *MAT2A* mRNA and protein that sensitized cancer stem cells to *MAT2A* inhibition by siRNAs or cycloleucine. The combination of dietary methionine restriction and cycloleucine was effective in suppressing primary and lung metastatic tumor burden in a murine TNBC model. SAM biosynthesis is a unique target for drug-resistant cancer stem cells [21].

Glycine N-methyltransferase (GNMT) catalyzes the transfer of a methyl group from SAM to S-adenosylhomocysteine (SAH), which is subsequently converted to the amino acid homocysteine by removal of the adenosine base [22,23]. We previously demonstrated that GNMT facilitates transmethylation kinetics, SAM homeostasis, and assists the conservation of methyl groups by limiting homocysteine remethylation/transsulfuration fluxes [24]. In addition to the regulation of methyl group availability, we also demonstrated that GNMT expression improves folate retention and bioavailability in the liver, assists methylfolate-dependent reactions, and ameliorates the consequences of folate depletion [25]. Our previous studies gave underlying mechanisms by which GNMT can participate in tumor prevention/suppression in humans [22,26].

GNMT may have distinct roles in different types of cancers. GNMT is commonly diminished in human liver cancers and is undetectable in cancer cell lines. GNMT nuclear localization was associated with induction of apoptosis that is independent of its catalytic activity or folate binding [22]. Overexpression of GNMT enhances nucleotide biosynthesis and improves DNA integrity by reducing uracil misincorporation in DNA both in vitro and in vivo [22,26]. On the other hand, siRNA-mediated *GNMT* knockdown results in an inhibition of proliferation, and induces G1 arrest and apoptosis in prostate cancer cell lines. Hence GNMT may play an important role in promoting prostate cancer cell growth via the regulation of apoptosis, and serve as a marker of malignant progression and poor prognosis of prostate cancer [23].

Expressions of sarcosine metabolism-related proteins including GNMT varied according to subtype of breast cancer [27]. Tissue microarray revealed that GNMT expression was higher in the androgen receptor (AR)-positive group compared with those of the AR-negative group [28]. HER-2 type tumors exhibited elevated expression of sarcosine metabolism-related proteins including GNMT, whereas TNBC subtype showed decreased expression. Expression of sarcosine metabolism-related proteins was associated with breast cancer prognosis. GNMT expression was found to be an independent factor for shorter disease-free survival [29]. In metastatic breast cancer, expression of GNMT is predominantly observed in brain and lung metastases [30]. The above studies point out an important role of GNMT in tumor initiation via methionine cycle flux, yet the role of GNMT in breast cancer is not fully elucidated.

Cancer cells within tumors are heterogeneous and dynamic. Proteome-wide mass spectrometry profiling revealed that MAT2A is among the cell cycle-dependent translocating proteins. Further analyses indicated that MAT2A may translocate to the nucleus after the G1/S-checkpoint, which enables epigenetic histone methylation maintenance during DNA replication [31]. This study pointed out a significant role of MAT2A in cell cycle and possibly cell proliferation.

In the present study, we explored the clinical significance of methionine cycle genes, including *GNMT*, *MAT1A* and *MAT2A* mRNA levels in breast carcinoma using RNA-seq data from the TCGA/GTEx datasets within GEPIA2, and further validated the findings in our own breast cancer cohort by immunohistochemistry. GNMT, MAT1A, MAT2A-biomarker IHC panel was compared with the clinical survival record in breast cancer patients, to examine the accuracy of IHC-based methods for identifying clinical prognosis.

## 2. Results

### 2.1. Identification of mRNA Expression of the Methionine Cycle Genes Signature for Survival in TGCA Dataset

**Figure 1:**
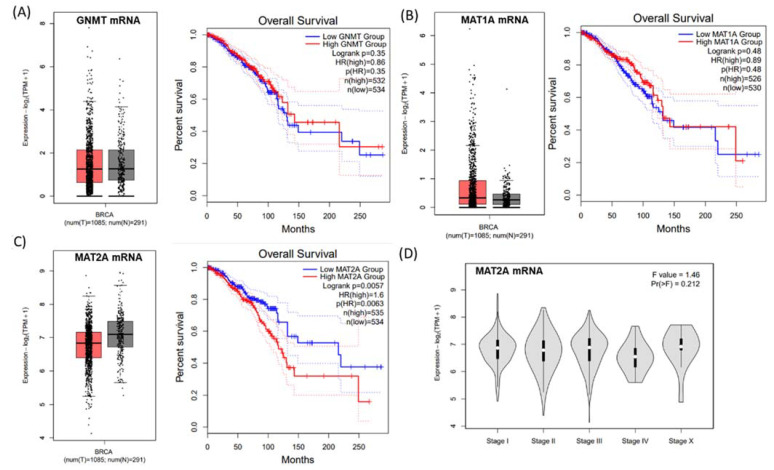
*GNMT*, *MAT1A* and *MAT2A* mRNA expression from a published RNA-seq data set. (**A**) No difference was observed in GNMT (**A**) or MAT1A (**B**) mRNA expressions between breast cancer tissues and normal tissues, and no association was found in these genes with overall survival rate. (**C**) The median of MAT2A mRNA expression level in breast tumorous tissues tended to be lower in breast cancer compared to that of the normal tissues; however, a longer survival rate was found in patients with lower MAT2A mRNA levels of the tumor tissue, (**D**) No association was found between MAT2A mRNA expression and tumor stages.

*GNMT*, *MAT1A* and *MAT2A* mRNA expression of the methionine cycle genes were verified by the GEPIA web tool (http://gepia2.cancer-pku.cn/#index, accessed on 1 May 2021) (Figure 1). mRNA expression of *GNMT* and *MAT1A* was not related to breast cancer survival (Figure 1A,B). A distinctly different expression pattern of *MAT2A* was observed between the breast tumor and normal samples: when compared to the normal breast tissues, the median of *MAT2A* mRNA expression level in breast tumorous tissues tended to be lower (Figure 1C). However, higher mRNA expression of *MAT2A* was significantly associated with poor survival in breast cancer patients (*p* = 0.0057, Figure 1C). There was no significant correlation between *MAT2A* mRNA expression and the tumor stage through the GEPIA database analyses (Figure 1D). The correlation of higher *MAT2A* expression with poorer survival was somewhat contradictory to the lower *MAT2A* mRNA expression pattern in the tumor tissues, hence we further aimed to investigate the role of MAT2A protein in more depth.

### 2.2. Expression of GNMT Is Downregulated, MAT1A Is Upregulated, and Nuclear MAT2A Is Downregulated in Breast Cancer Tissues

**Figure 2:**
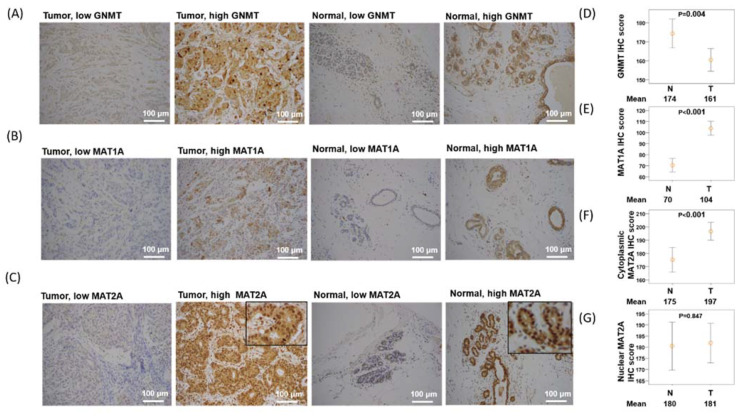
GNMT, MAT1A, and MAT2A immunohistochemical staining in selected cases of breast cancer (×200). (**A**) Low and high GNMT staining in breast cancer tissues and normal breast tissues. (**B**) Low and high MAT1A staining in breast cancer tissues and normal breast tissues. (**C**) Low and high MAT2A staining in breast cancer tissues and normal breast tissues. (**D**) GNMT is overexpressed in normal tissues versus breast cancer tissues. (**E**) MAT1A is overexpressed in breast cancer tissues versus normal tissues. (**F**) Cytoplasmic MAT2A is overexpressed in breast cancer tissues versus normal tissues. (**G**) No difference is observed in nuclear MAT2A expression between breast cancer tissues versus normal tissues.

The nucleus translocation of MAT2A has been proposed to enable epigenetic histone methylation maintenance during DNA replication in vitro [32]. The previous in vitro finding of MAT2A translocation in cancer cell lines as well as our findings on MAT2A mRNA and breast cancer survival inspired us to explore the prognostic potential and the clinical application of the subcellular localization of MAT2A in our breast cancer cohort study. We examined the specimens from 252 independent patients and compared the subcellular protein expression of GNMT, MAT1A, and MAT2A between the breast tumor and their paired normal breast tissues by Immunohistochemistry (IHC) analysis. The clinicopathological data are presented in Table 1, and representative IHC staining for each target molecule is shown in Figure 2A–C. The GNMT and MAT1A proteins were found exclusively in the cytoplasmic fraction of tumor and normal tissues (Figure 2A,B). In contrast, MAT2A protein were found in both nuclear (N) and cytoplasmic (C) fractions of the tumor and normal tissues (Figure 2C). IHC analysis revealed that GNMT was downregulated in breast tumor tissues compared with normal breast tissues (*p* = 0.004, Figure 2D). On the other hand, MAT1A was upregulated in breast cancerous tissues compared with normal breast tissues (*p* < 0.001, Figure 2E). Furthermore, cytoplasmic MAT2A was upregulated in breast cancer tissues compared with normal breast tissues (*p* < 0.001, Figure 2F). No statistical difference was found in nuclear MAT2A expression between normal and breast cancer tissues (Figure 2G).

IHC staining of tissue microarray discovered that a higher cytoplasmic/nuclear (C/N) ratio of MAT2A protein was observed in more (63.0%, 29/46) patients aged above 65 (*p* = 0.050, Table 1). On the other hand, low MAT1A protein expression was observed in more (63.0%, 29/46) patients aged above 65 (*p* = 0.050, Table 1).

### 2.3. Identification of a Gene Expression Signature for Survival

**Figure 3:**
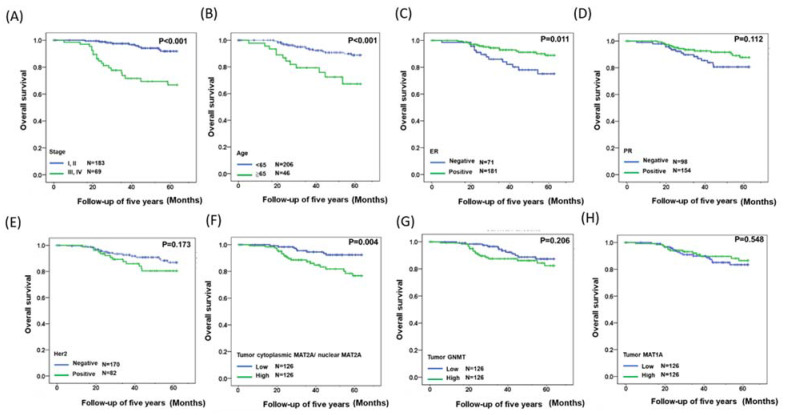
Kaplan–Meier analysis of clinical data, GNMT, MAT1A, and C/N ratio of MAT2A protein expressions in breast cancer patients. (**A**) Overall survival estimates for stage. (**B**) Overall survival estimates for age. (**C**) Overall survival estimates for ER expression. (**D**) Overall survival estimates for PR expression. (**E**) Overall survival estimates for HER2 expression. (**F**) Overall survival estimates for C/N ratio of MAT2A expression. (**G**) Overall survival estimates for GNMT expression. (**H**) Overall survival estimates for of MAT1A expression.

The GNMT and MAT1A protein expressions and the C/N ratio of MAT2A were also correlated with the five-year relative survival rate in our breast cancer cohort. Kaplan–Meier survival analyses were performed after the samples were classified into high- and low-expression groups according to the median scores, and stage status was used to evaluate the prognosis of the patients within this period [33]. As shown in Figure 3A, patients in stage III and IV tumors had a poorer survival than those in stage I and II (*p* < 0.001). Patients aged 65 and above were associated with a significant increase in 5 years mortality rate compared with those aged below 65 (*p* < 0.001, Figure 3B). Patients with positive expression of ER were associated with significantly improved breast cancer survival rate compared with those with negative ER (*p* < 0.001, Figure 3C). Higher C/N ratio of MAT2A in the tumorous tissues was associated with poorer survival (*p* = 0.004, Figure 3F). Neither GNMT (Figure 3G) nor MAT1A (Figure 3H) protein expression was associated with patient survival rate in our cohort.

Furthermore, multivariate logistic-regression analysis indicated that a higher MAT2A C/N ratio significantly correlated with poorer survival (hazard ratio = 2.771, 95% confidence interval (CI) 1.186–6.472) (Table 2). Taken together, high C/N ratio of MAT2A in the tumorous tissues of breast cancer patients is associated with poor prognosis that is independent of age, ER, and tumor-node-metastasis (TNM) stages.

**Figure 4:**
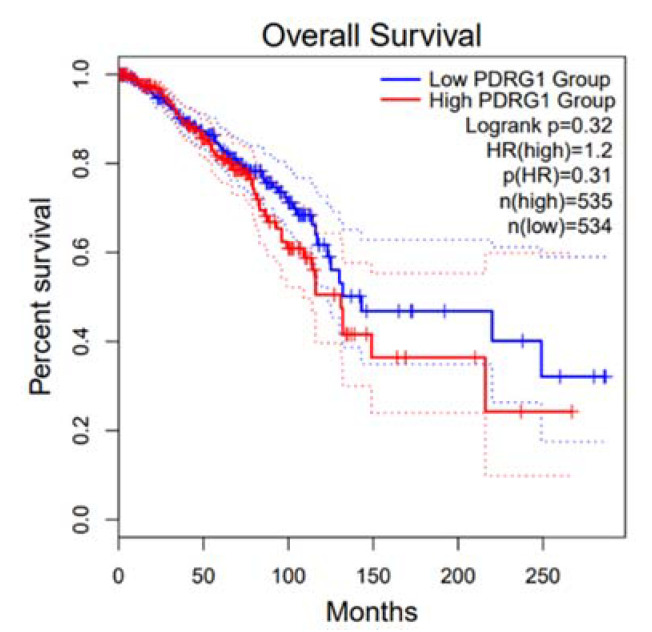
Kaplan–Meier analysis of *PDRG1* mRNA.

In addition to the potential epigenetic regulation of histone, MAT2A may affect cancer progression via its interactions with other nuclear proteins. The oncogene *P53 and DNA Damage Regulated 1 (PDRG1)* encoded protein PDRG1 has been reported as an interaction target of methionine adenosyltransferases in the control of the nuclear methylation status [34]. Therefore, we further examined the clinical relevance of PDG1 in breast cancer. Kaplan–Meier analysis using the dataset in GEPIA indicated no significant correlation between *PDRG1* mRNA expression and breast cancer patient survival (Figure 4).

### 2.4. Localization of MAT2A in Breast Cancer Cell Lines and the Association of MAT2A C/N Ratio and Cell Invasiveness

**Figure 5:**
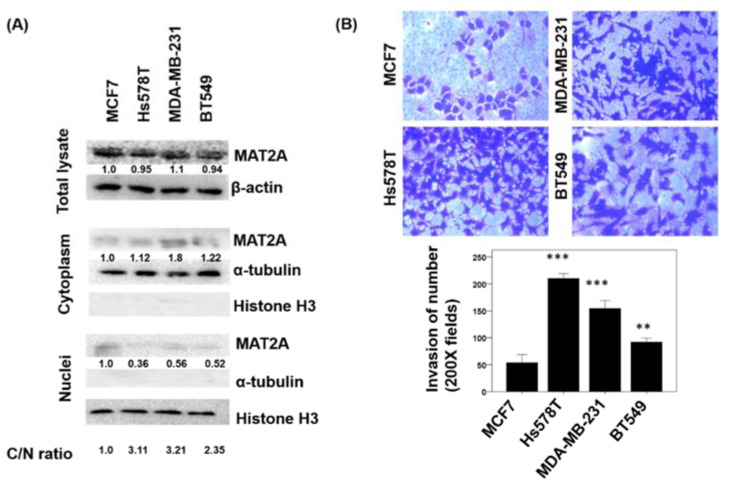
Cellular protein expression pattern of MAT2A, and the invasiveness were investigated in a panel of breast cancer cell lines. (**A**) Cytoplasmic and nuclear protein lysates were prepared from the MCF7, Hs578T, MDA-MB-231, and BT549 breast cancer cell lines and protein expression levels were analyzed by Western blotting using specific antibodies against MAT2A, β-actin, α-tubulin, and Histone H3. (**B**) Invasion assay in MCF7, Hs578T, MDA-MB-231, and BT549 breast cancer cell lines. C/N ratio: cytoplasm to nucleus ratio. The histogram represents means ± SEs from three independent experiments (**, *p* < 0.01; ***, *p* < 0.001).

To investigate MAT2A subcellular localization in human breast cancer cells and explore whether they are potentially involved in cancer cell invasion, we compared the MAT2A protein levels in the cytoplasm and nucleus (Figure 5A) as well as the invasiveness (Figure 5B) in a panel of breast cancer cell lines. MAT2A protein were detected in both the cytoplasm and nucleus of MCF7, Hs578T, MDA-MB-231, and BT549. Protein quantification and MAT2A C/N ratio values were calculated by Image J and are shown in Figure 5A. Our data indicated that a higher MAT2A C/N and/or lower nuclear MAT2A expression may be related to increased invasiveness. Cell lines with higher MAT2A C/N (Hs578T andMDA-MB231) were more invasive, whereas MCF7 that had the lowest C/N ratio were the least invasive (Figure 5B).

## 3. Discussion

Folate and folate-mediated one-carbon metabolism play a crucial role in human cancer development and therapeutics [14,34]. MAT2B expression has been reported to correlate with poor prognosis in TNBC and targeting MAT2B was proposed to be a potential therapeutic target for TNBC [19]. However, MAT2A has not been elucidated in breast cancer prognosis. Combining mRNA gene expression data from public datasets, IHC protein expression pattern of tissue array from a cohort study, and cancer cell invasion data from breast cell lines, the present study provides a novel prognostic marker for breast cancer development. We successfully demonstrated that the subcellular distribution (C/N ratio) of a key methionine cycle enzyme, MAT2A, can predict a poorer survival in breast cancer patients.

Methionine cycle enzymes have been found to be enriched in numerous tumor types, and MAT2A expression impinges upon the sensitivity of certain cancer cells to therapeutic inhibition. Metabolomics and metabolite tracing analyses revealed that tumor-initiating cells in the lung have highly elevated methionine cycle activity and transmethylation rates that are driven by MAT2A. Inhibition of the methionine cycle impeded the tumor-initiating capability of these cells [16]. High methionine cycle activity increased methionine consumption and made the cells to be dependent on exogenous methionine [16]. MAT2A inhibition was found to disrupt the tumorigenicity of lung tumor-initiating cells, which led to a decrease in histone methylation [16]. Inhibition of MAT2A significantly suppressed HCC cell growth at the G1/S phase and the expressions of p21, p27, and bax [35]. On the other hand, MAT2A expression was lower in the tumor tissues of human renal cell carcinomas (RCC) [36], suggesting that MAT2A may have a potential role in the development of RCC. Whether these mechanisms are involved in breast cancer remains to be studied further.

Our study is the first one to investigate the clinical prognosis potential of MAT2A in breast cancer. Using the GEPIA, a poorer breast cancer survival was observed in patients with higher *MAT2A* mRNA level, suggesting a potential role of MAT2A in breast cancer development. Previous in vitro cell cycle profiling in Hela cells revealed that the translocation of MAT2A to the nucleus occurred after G1/S checkpoint, which enabled epigenetic histone methylation during DNA replication on cell cycle dynamics [31]. Despite the potential regulatory role of MAT2A translocation in tumor development, whether MAT2A distribution affect in breast cancer progression is unknown, thus we aimed to explore the relationship between MAT2A distribution and breast cancer clinical indicators. Since the actual distributions and localizations of MAT2A protein cannot be determined in RNA-seq data, we further investigated the prognostic potential of MAT2A protein distributions and localizations in our own breast cancer cohort using tissue array. A MAT2A immunoreactivity was observed in the cytoplasm and nuclei in the breast cancer and adjacent normal tissue. Interestingly, patients with higher C/N MAT2A ratios had lower 5 year survival rates than those with lower C/N ratios. Multivariate Cox regression model analysis further validated the independent prognostic role of MAT2A when grouped by C/N ratio.

The cause of increased *MAT2A* expression in breast cancer cells is of interest. In HCC cells, increased transactivation of NF-kappa B and AP-1 contributes to *MAT2A* upregulation [37]. Nuclear binding of NF-kappa B and AP-1 to the *MAT2A* promoter are increased in HCC, and tumor necrosis factor alpha (TNFα), which activates both sites, can increase *MAT2A* expression in a dose- and time-dependent manner [37]. TNF-α levels have been found to be correlated with clinical disease stage and lymph node metastasis, as well as with ER and HER2 antigen expression in breast cancer patients [38]. Hence, it is plausible that the elevated TNF-α partially accounts for the induction and overexpression of *MAT2A* in breast cancer, and there could be a link between inflammation and *MAT2A* expression during breast cancer progression. Previously, we discovered in vitro and in vivo evidence that low-dose anti-inflammatory DMARD methotrexate inhibits MAT genes, proteins, and enzyme activity [39] and thus raised concerns about perturbed methylation reactions in humans on low-dose methotrexate for treating rheumatoid arthritis. Future studies on the clinical physiological consequences of MAT inhibition, SAM supply in breast cancer are warranted.

MAT2A may modulate human disease pathogenesis via SAM supply. Carbon tetrachloride-induced *MAT2A* overexpression facilitates mouse hepatic fibrosis through the regulation of intracellular SAM concentration [40]. Transforming growth factor β1 (TGF-β1) induces the activation of NF-κB that promotes mRNA and protein expression of MAT2A and reduces SAM concentration in hepatic stellate cells [41], suggesting that the action of MAT2A in human pathogenesis might involve SAM homeostasis. SAM, as a universal methyl donor, has been proposed to be involved in chemoprevention and chemotherapy. SAM is anti-apoptotic in normal hepatocytes but pro-apoptotic in liver cancer cells. In liver cancer cells but not in normal human hepatocytes, SAM can selectively induce Bcl-x(S), an alternatively spliced isoform of Bcl-x(L) that promotes apoptosis. This makes SAM an ideal candidate agent for both chemoprevention and treatment of HCC [41]. Furthermore, MAT2A was reported to act as a transcriptional corepressor for heme oxygenase-1 (HO-1) expression by supplying SAM for methyltransferases, thus it was suggested to act as a tumor suppressor in kidney carcinogenesis [39]. In vitro proteomics indicated that MATII serves as a transcriptional corepressor of oncoprotein MafK by interacting with chromatin regulators and supplying SAM for methyltransferases [42]. MAT2A protein may provide SAM locally on chromatin where it interacts with many chromatin-associated proteins with various functions including histone modification, epigenic remodeling, transcription regulation, and nucleo-cytoplasmic transfer [43].

MAT2A protein is involved in methyl donor production and was previously found to have a dynamic nuclear localization, and whether MAT2A protein localization may influence breast cancer development is unknown. In human liver cancer, nuclear MATα interacts physically and functionally with an onco-protein PDRG1 (P53 and DNA Damage Regulated 1) that leads to reduced DNA methylation. Increased PDRG1 expression is detected in acute liver injury and hepatoma cells, together with decreased MAT1A expression and nuclear accumulation of MATα1. Silencing of PDRG1 in hepatoma cells downregulates genes associated with tumor progression according to GO pathway analysis. Yeast two hybrid and rat liver library revealed that onco-protein PDRG1 is an interacting target of MATs [32]. These data indicated that PDRG1 is involved in the progression of hepatic diseases by controlling the nuclear methylation through binding with MAT enzyme [32]. The binding of methionine adenosyltransferase and its putative collaboration with PDRG1 was proposed to control of the nuclear methylation status in HCC; we therefore explored the possible role of PDRG1 in breast cancer. However, Kaplan–Meier survival analyses using RNA-seq data from the GEPIA indicated that *Pdrg1* gene expression is not related to breast cancer survival. No association was observed between overall survival and mRNA expression levels of *PDRG1* (Figure 4).

GNMT was downregulated in breast tumor tissues compared with normal breast tissues. GNMT has been proposed to be a novel tumor suppressor in cellular defense against DNA damage [22]. Conversely, the increased MAT1A in the breast tumor compared to the control tissues implied a potential role of oncogene that may deserve attention in future studies. Nevertheless, neither GNMT nor MAT1A protein expression was associated with patient survival rate in our cohort.

Many transformed cells rely on *MAT2A* to synthesize SAM and maintain their epigenome. Higher level of *MAT2B* has been found to be correlated with worse relapse-free survival in the TNBC [19]. *MAT2B* encodes a β-subunit without catalytic action, but it can regulate MATII enzymatic activity [15]. Induction of *MAT2A/MAT2B* favors tumor growth and survival and also enhances tumor migration [15]. Direct inhibition of *MAT2B* suppressed cell growth and migration and induced apoptosis in breast cancer cell MDA-MB-231 and MDA-MB-468 [19]. Future studies on how MAT2A localization may modulate breast cancer development and progression are warranted.

## 4. Materials and Methods

### 4.1. Web Server Survival Analysis

The expression analysis of *GNMT*, *MAT1A* and *MAT2A* mRNA in breast tumor and breast normal tissues was calculated using ANOVA. The Kaplan–Meier survival analysis of GNMT, MAT1A, and MAT2A mRNA expression was performed on the BRCA RNA-seq data of the TCGA/GTEx datasets available within GEPIA2, by autoselecting the median values between the lower and upper quartiles into high and low expression. More information can be found at http://gepia2.cancer-pku.cn/#index (accessed on 25 October 2020) [44].

### 4.2. Patients

Contralateral primary breast tumor and adjacent normal breast tissues of 252 breast cancer patients receiving surgical resection were acquired from Changhua Show Chwan Memorial Hospital from March 2011 to January 2017. Computed tomography (CT) was applied for diagnosis in the 265 breast cancer patients prior to surgery. The diagnosis parameters and clinical outcomes were recruited until patient death or loss to follow-up. The age of all patients was between 29 and 95 years (mean ± SD = 54.88 ± 12.32). Clinical parameters and survival data were recorded from the cancer registry system of Changhua Show Chwan Memorial Hospital. Survival data was annotated to be the following time from the date of primary surgery to the date of death. During this survey, 30 patients died and 42 patients exhibited tumor metastasis, with the metastasis sites, including skin, abdomen, pleura, bone, lung, liver, chest wall, breast, and lymph node. The median overall survival of all breast cancer patients was 48 months. This project was approved by the Ethics Committee of the Institutional Review Board of Show Chwan Memorial Hospital (IRB No. 1060407, 7 April 2017).

### 4.3. Immunohistochemistry and Scoring

For each patient, representative tissue cores of the BC tumor section as well the adjacent normal section were carefully collected and made into tissue microarray. Immunohistochemistry (IHC) staining was used to evaluate GNMT, MAT1A, and MAT2A protein expression. The GNMT antibody (Proteintech, 18790-1-AP) was purchased from Proteintech Group, Inc. (Rosemont, IL 60018, USA). MAT1A antibody (Novus, NBP2-33533) was purchased from Novus Biologicals, LLC, Inc. (Centennial, CO 80112, USA)., and MAT2A antibody (GTX50027; GeneTex) was purchased from GeneTex, Inc. (Alton Pkwy Irvine, CA, USA). IHC evaluation and protocol were used to obtain score have been descripted previously [45,46]. The mean signals scores were evaluated independently by the two pathologists who were blinded when assessing the samples. Immunostaining scores were defined as the cell staining intensity (0 = nil; 1 = weak; 2 = moderate; and 3 = strong) multiplied by the percentage of labeled cells (0% to 100%), leading to scores from 0 to 300. The mean of score of signals were evaluated independently by the two pathologists. Immunostaining scores were defined as the cell staining intensity (0 = nil; 1 = weak; 2 = moderate; and 3 = strong) and multiplied by the percentage of labelled cells (0% to 100%), leading to scores ranging from 0 to 300. The median IHC staining median score was used as the cutoff point for the dichotomization of GNMT, MAT1A, and C/N ratio of MAT2A. A score greater median score was defined as “high” immunostaining, whereas a score of less or equal than median score was defined as “low.”

### 4.4. Cell Culture

The human breast cancer cells were purchased from the American Type Culture Collection (ATCC, Gaithersburg, MD, USA). The human breast cancer MCF7, Hs578T, MDA-MB-231, and BT549 cells were cultured in low glucose Dulbecco’s Modified Eagle’s Medium (DMEM) supplemented with 10% FBS (HyClone, Logan, UT, USA). T47D and BT549 were grown in RPMI 1640 (Corning, NY, USA) with 10% FBS. Cells were maintained in a humidified incubator with 5% CO_2_ at 37 °C.

### 4.5. Western Blot Analysis

The cells were harvested using a curet and centrifuged at 1000× *g* for 10 min at 4 °C and then lysed in ice-cold radioimmunoprecipitation assay (RIPA) lysis buffer (Catalog number: 89900, Thermo Scientific company, Waltham, MA, USA) with 100 µL protease inhibitor cocktail (Roche, Pleasanton, CA, USA). Equal amounts of protein (30 µg) were separated by SDS-PAGE (10% gel) and subsequently transferred to a polyvinylidene difluoride membrane. Subsequent to blocking with 5% skimmed milk at room temperature for 1 h, the membranes were incubated at 4 °C overnight with primary antibodies, including anti-MAT2A (1:1000; GTX50027; GeneTex), anti-β-actin (1:500; tcea13161; TAICLONE BIOTECH CORP.), anti-α-tubulin (1:500; GTX112535; GeneTex), anti-Histone H3 (1:1,000; #3932; GTX122148; GeneTex), followed by incubation at room temperature for 2 h with HRP-conjugated polyclonal secondary antibody (1:5000; GTX213110-01/GTX213111-01; GeneTex). All Western blots were visualized using the enhanced plus chemiluminescence assay kit (EMD Millipore, Billerica, MA, USA), according to the manufacturer’s protocol. Protein expression levels in cells were quantified by ImageJ software (https://imagej.nih.gov/ij/, accessed on 1 May 2021).

### 4.6. Transwell Invasion Assay

Cell invasion was investigated using Matrigel invasion chambers with a pore size of 8 μm (Costar; Corning Life Sciences, Cambridge, MA, USA). Briefly, MCF7, Hs578T, MDA-MB-231, and BT549 cells (4 × 10^4^ cells per chamber) in serum-free medium were seeded in the upper chamber, and 10% fetal bovine serum (FBS) (Gibco; Thermo Fisher Scientific, Inc., Waltham, MA, USA) was used as a chemoattractant in the bottom well. After incubation for 24 h at 37 °C, the non-invasive cells on the upper surface of the membrane were removed with a cotton swab, and the invasive cells on the bottom side were fixed in 100% methanol at room temperature for 5 min, stained with 1% crystal violet at room temperature for 10 min and counted using a microscope (Nikon Eclipse80i; Nikon Corporation, Melville, NY, USA) under ×200 magnification with five fields of view per cells.

### 4.7. Statistical Analysis

The association between GNMT, MAT1A, and MAT2A protein expression and the clinical and pathological parameters was calculated using Chi-square and paired-sample t-tests, and survival curves were plotted using the Kaplan–Meier method and compared using log-rank test. Cox’s proportional hazards regression model was used to analyze the association between the variables and survival data. *p* < 0.05 was considered to indicate a statistically significant difference. by SPSS 18.0 (SPSS, Inc., Chicago, IL, USA) was used for all statistical analyses.

## 5. Conclusions

The present study demonstrated a novel strategy that used the MAT2A C/N ratio rather than the MAT2A expression for breast cancer prognosis. Furthermore, it is noteworthy that high C/N ratio (>1) of MAT2A protein expression was present in more than 50% of the breast cancer specimens in our cohort. In vitro studies found that breast cancer cell lines with a higher MAT2A C/N ratio were more invasive. MAT2A C/N expression ratio determined by IHC staining could serve as a novel independent prognostic marker for breast cancer. The modulation of MAT2A subcellular localization and function may serve as a potential novel therapeutic strategy for breast cancer.

## Figures and Tables

**Table 1:** Relationships between clinical parameters, GNMT1, MAT1A protein expression, and MAT2A C/N ratio in 252 breast cancer patients.

		GNMT1			MAT1A			MAT2A (C/N Ratio)		
Characteristics	No.	Low (N = 126)	High (N = 126)	*p*-Value	Low (N = 126)	High (N = 126)	*p*-Value	Low (N = 126)	High (N = 126)	*p*-Value
Age										
<65	206	99 (48)	107 (52)	0.19	97 (47)	109 (53)	0.05	109 (53)	97 (47)	0.05
≥65	46	27 (59)	19 (41)		29 (63)	17 (37)		17 (37)	29 (63)	
Stage										
I, II	183	52 (28)	131 (72)	0.89	72 (39)	111 (61)	0.81	95 (52)	88 (48)	0.323
III, IV	69	19 (28)	50 (72)		26 (38)	43 (62)		31 (45)	38 (55)	
ER										
Negative	71	37 (52)	34 (48)	0.67	34 (48)	47 (52)	0.67	37 (52)	34 (48)	0.674
Positive	181	89 (49)	92 (51)		92 (51)	89 (49)		89 (49)	92 (51)	
PR										
Negative	98	54 (55)	44 (45)	0.2	50 (51)	48 (49)	0.8	49 (50)	49 (50)	1
Positive	154	72 (47)	82 (53)		76 (49)	78 (51)		77 (50)	77 (50)	
HER2										
Negative	170	82 (48)	88 (52)	0.42	84 (49)	86 (51)	0.79	89 (52)	81 (48)	0.282
Positive	82	44 (54)	38 (46)		42 (51)	40 (49)		37 (45)	45 (55)	

**Table 2:** Results of multivariate Cox regression model for age, stage, and C/N ratio of MAT2A expression.

Characteristics	(Favorable/Unfavorable)	HR	95.0% CI	*p*-Value
Age	<65/≥65	3.730	1.717–8.101	0.004
ER	Negative/Positive	4.442	2.002–9.855	<0.001
Stage	I, II/III, IV	8.276	3.627–18.884	<0.001
MAT2A (C/N ratio)	Low/High	2.771	1.186–6.472	0.018

## Data Availability

The data presented in this study are available on request from the corresponding author.

## References

[1] Siegel R.L., Miller K.D., Jemal A. (2019). Cancer statistics, 2019. CA Cancer J. Clin..

[2] DeSantis C.E., Ma J., Gaudet M.M., Newman L.A., Mph K.D.M., Sauer A.G., Jemal A., Siegel R.L. (2019). Breast cancer statistics, 2019. CA Cancer J. Clin..

[3] DeSantis C.E., Ma J., Sauer A.G., Newman L.A., Jemal A. (2017). Breast cancer statistics, 2017, racial disparity in mortality by state. CA Cancer J. Clin..

[4] Youlden D.R., Cramb S.M., Yip C.H., Baade P.D. (2014). Incidence and mortality of female breast cancer in the Asia-Pacific region. Cancer Biol. Med..

[5] Koren S., Bentires-Alj M. (2015). Breast Tumor Heterogeneity: Source of Fitness, Hurdle for Therapy. Mol. Cell.

[6] Januškevičienė I., Petrikaitė V. (2019). Heterogeneity of breast cancer: The importance of interaction between different tumor cell populations. Life Sci..

[7] Rivenbark A.G., O’Connor S.M., Coleman W.B. (2013). Molecular and cellular heterogeneity in breast cancer: Challenges for personalized medicine. Am. J. Pathol..

[8] Palma G., Frasci G., Chirico A., Esposito E., Siani C., Saturnino C., Arra C., Ciliberto G., Giordano A., D’Aiuto M. (2015). Triple negative breast cancer: Looking for the missing link between biology and treatments. Oncotarget.

[9] Wang Y.C., Wu M.T., Tang F.Y., Chen D.Y., Ko H.A., Shane B., Huang W.-N., Chiang E.-P.I. (2019). MTHFR C677T polymorphism increases MTX sensitivity via the inhibition of S-adenosylmethionine and de novo purine synthesis. Clin. Sci..

[10] Anderson D.D., Woeller C.F., Chiang E.P., Shane B., Stover P.J. (2012). Serine hydroxymethyltransferase anchors de novo thymidylate synthesis pathway to nuclear lamina for DNA synthesis. J. Biol. Chem..

[11] Tan Y.L., Sou N.L., Tang F.Y., Ko H.A., Yeh W.T., Peng J.H., Chiang E.P.I. (2020). Tracing Metabolic Fate of Mitochondrial Glycine Cleavage System Derived Formate In Vitro and In Vivo. Int. J. Mol. Sci..

[12] Sou N.L., Huang Y.H., Chen D.Y., Chen Y.M., Tang F.Y., Ko H.A., Fan Y.H., Lin Y.Y., Wang Y.C., Chih H.M. (2021). Folinate Supplementation Ameliorates Methotrexate Induced Mitochondrial Formate Depletion In Vitro and In Vivo. Int. J. Mol. Sci..

[13] Stempak J.M., Sohn K.J., Chiang E.P., Shane B., Kim Y.I. (2005). Cell and stage of transformation-specific effects of folate deficiency on methionine cycle intermediates and DNA methylation in an in vitro model. Carcinogenesis.

[14] Chiang E.P., Wang Y.C., Tang F.Y. (2007). Folate restriction and methylenetetrahydrofolate reductase 677T polymorphism de-creases adoMet synthesis via folate-dependent remethylation in human-transformed lymphoblasts. Leukemia.

[15] Maldonado L.Y., Arsene D., Mato J.M., Lu S.C. (2017). Methionine adenosyltransferases in cancers: Mechanisms of dysregulation and implications for therapy. Exp. Biol. Med..

[16] Wang Z., Yip L.Y., Lee J.H.J., Wu Z., Chew H.Y., Chong P.K.W., Teo C.C., Ang H.Y.-K., Peh K.L.E., Yuan Y. (2019). Methionine is a metabolic dependency of tumor-initiating cells. Nat. Med..

[17] Tomasi M.L., Cossu C., Spissu Y., Floris A., Ryoo M., Iglesias-Ara A., Wang Q., Pandol S.J., Bhowmick N.A., Seki E. (2017). S-adenosylmethionine and methylthioadenosine inhibit cancer metastasis by targeting microRNA 34a/b-methionine adenosyltransferase 2A/2B axis. Oncotarget.

[18] Simile M.M., Peitta G., Tomasi M.L., Brozzetti S., Feo C.F., Porcu A., Cigliano A., Calvisi D.F., Feo F., Pascale R.M. (2019). MicroRNA-203 impacts on the growth, aggressiveness and prognosis of hepatocellular carcinoma by targeting MAT2A and MAT2B genes. Oncotarget.

[19] Xu J., Wu D., Wang S., Wang Z. (2019). MAT2B expression correlates with poor prognosis in triple-negative breast cancer. Cancer Manag. Res..

[20] Phuong N.T., Kim S.K., Im J.H., Yang J.W., Choi M.C., Lim S.C., Lee K.Y., Kim Y.M., Yoon J.H., Kang K.W. (2016). Induction of methionine adenosyltransferase 2A in tamoxifen-resistant breast cancer cells. Oncotarget.

[21] Strekalova E., Malin D., Weisenhorn E.M.M., Russell J.D., Hoelper D., Jain A., Coon J.J., Lewis P.W., Cryns V.L. (2019). S-adenosylmethionine biosynthesis is a targetable metabolic vulnerability of cancer stem cells. Breast Cancer Res. Treat..

[22] Wang Y.C., Lin W.L., Lin Y.J., Tang F.Y., Chen Y.M., Chiang E.P. (2014). A novel role of the tumor suppressor GNMT in cellular defense against DNA damage. Int. J. Cancer.

[23] Song Y.H., Shiota M., Kuroiwa K., Naito S., Oda Y. (2011). The important role of glycine N-methyltransferase in the carcinogenesis and progression of prostate cancer. Mod. Pathol..

[24] Wang Y.C., Tang F.Y., Chen S.Y., Chen Y.M., Chiang E.P. (2011). Glycine-N methyltransferase expression in HepG2 cells is in-volved in methyl group homeostasis by regulating transmethylation kinetics and DNA methylation. J. Nutr..

[25] Wang Y.C., Chen Y.M., Lin Y.J., Liu S.P., Chiang E.P. (2011). GNMT expression increases hepatic folate contents and folate-dependent methionine synthase-mediated homocysteine remethylation. Mol. Med..

[26] Wang Y.C., Wu M.T., Lin Y.J., Tang F.Y., Ko H.A., Chiang E.P. (2015). Regulation of Folate-Mediated One-Carbon Metabolism by Glycine N-Methyltransferase (GNMT) and Methylenetetrahydrofolate Reductase (MTHFR). J. Nutr. Sci. Vitaminol..

[27] DebRoy S., Kramarenko I.I., Ghose S., Oleinik N.V., Krupenko S.A., Krupenko N.I. (2013). A novel tumor suppressor function of glycine N-methyltransferase is independent of its catalytic activity but requires nuclear localization. PLoS ONE.

[28] Kim M.J., Jung W.H., Koo J.S. (2015). Expression of sarcosine metabolism-related proteins in estrogen receptor negative breast cancer according to the androgen receptor and HER-2 status. Int. J. Clin. Exp. Pathol..

[29] Yoon J.K., Kim D.H., Koo J.S. (2014). Implications of differences in expression of sarcosine metabolism-related proteins according to the molecular subtype of breast cancer. J. Transl. Med..

[30] Cha Y.J., Kim D.H., Jung W.H., Koo J.S. (2014). Expression of sarcosine metabolism-related proteins according to metastatic site in breast cancer. Int. J. Clin. Exp. Pathol..

[31] Herr P., Boström J., Rullman E., Rudd S.G., Vesterlund M., Lehtiö J., Helleday T., Maddalo G., Altun M. (2020). Cell Cycle Profiling Reveals Protein Oscillation, Phosphorylation, and Localization Dynamics. Mol. Cell. Proteom..

[32] Perez C., Perez-Zuniga F.J., Garrido F., Reytor E., Portillo F., Pajares M.A. (2016). The Oncogene PDRG1 Is an Interaction Target of Methionine Adenosyltransferases. PLoS ONE.

[33] Koo M.M., Swann R., McPhail S., Abel G.A., Elliss-Brookes L., Rubin G.P., Lyratzopoulos G. (2020). Presenting symptoms of cancer and stage at diagnosis: Evidence from a cross-sectional, population-based study. Lancet Oncol..

[34] Lee T.Y., Chiang E.P., Shih Y.T., Lane H.Y., Lin J.T., Wu C.Y. (2014). Lower serum folate is associated with development and invasiveness of gastric cancer. World J. Gastroenterol..

[35] Wang Q., Liu Q.-Y., Liu Z.-S., Qian Q., Sun Q., Pan D.-Y. (2008). Inhibition of hepatocelluar carcinoma MAT2A and MAT2beta gene expressions by single and dual small interfering RNA. J. Exp. Clin. Cancer Res..

[36] Wang X., Guo X., Yu W., Li C., Gui Y., Cai Z. (2014). Expression of methionine adenosyltransferase 2A in renal cell carcinomas and potential mechanism for kidney carcinogenesis. BMC Cancer.

[37] Yang H., Sadda M.R., Yu V., Zeng Y., Lee T.D., Ou X., Chen L., Lu S.C. (2003). Induction of human methionine adenosyltransferase 2A expression by tumor necrosis factor alpha. Role of NF-kappa B and AP-1. J. Biol. Chem..

[38] Ma Y., Ren Y., Dai Z.-J., Wu C.-J., Ji Y.-H., Xu J. (2017). IL-6, IL-8 and TNF-α levels correlate with disease stage in breast cancer patients. Adv. Clin. Exp. Med..

[39] Wang Y.C., Chiang E.-P. (2012). Low-dose methotrexate inhibits methionine S-adenosyltransferase in vitro and in vivo. Mol. Med..

[40] Wang K., Fang S., Liu Q., Gao J., Wang X., Zhu H., Zhu Z., Ji F., Wu J., Ma Y. (2019). TGF-beta1/p65/MAT2A pathway regulates liver fibrogenesis via intracellular SAM. EBioMedicine.

[41] Lu S.C., Mato J.M. (2008). S-Adenosylmethionine in cell growth, apoptosis and liver cancer. J. Gastroenterol. Hepatol..

[42] Katoh Y., Ikura T., Hoshikawa Y., Tashiro S., Ito T., Ohta M., Kera Y., Noda T., Igarashi K. (2011). Methionine adenosyltransferase II serves as a transcriptional corepressor of Maf oncoprotein. Mol. Cell.

[43] Igarashi K., Katoh Y. (2013). Metabolic Aspects of Epigenome: Coupling of S-Adenosylmethionine Synthesis and Gene Regulation on Chromatin by SAMIT Module. Subcell. Biochem..

[44] Tang Z., Kang B., Li C., Chen T., Zhang Z. (2019). GEPIA2: An enhanced web server for large-scale expression profiling and interactive analysis. Nucleic Acids Res..

[45] Chen Y.L., Lin P.Y., Ming Y.Z., Huang W.C., Chen R.F., Chen P.M., Chu P.Y. (2017). The effects of the location of cancer stem cell marker CD133 on the prognosis of hepatocellular carcinoma patients. BMC Cancer.

[46] Chu P.Y., Wang S.M., Chen P.M., Tang F.Y., Chiang E.P. (2020). Expression of MTDH and IL-10 Is an Independent Predictor of Worse Prognosis in ER-Negative or PR-Negative Breast Cancer Patients. J. Clin. Med..

